# Indirect Modeling of Post-Prandial Intestinal Lymphatic Uptake of Halofantrine Using PBPK Approaches: Limitations and Implications

**DOI:** 10.3390/pharmaceutics17091228

**Published:** 2025-09-22

**Authors:** Malaz Yousef, Farag E. S. Mosa, Khaled H. Barakat, Neal M. Davies, Raimar Löbenberg

**Affiliations:** Faculty of Pharmacy & Pharmaceutical Sciences, Katz Centre for Pharmacy & Health Research, University of Alberta, Edmonton, AB T6G 2E1, Canada; malaz@ualberta.ca (M.Y.); mosa@ualberta.ca (F.E.S.M.); kbarakat@ualberta.ca (K.H.B.)

**Keywords:** in silico, PBPK, lymphatic uptake, halofantrine, chylomicrons

## Abstract

**Background/Objectives:** Despite the recognized importance and distinctive characteristics of the intestinal lymphatic pathway in drug absorption, its pharmacokinetic modeling remains largely unexplored. This study aimed to address this gap by developing a physiologically based pharmacokinetic model (PBPK) to represent the oral lymphatic uptake of halofantrine following a fatty meal. **Methods**: Using GastroPlus™ 9.8.3 and published literature data, halofantrine absorption, distribution, metabolism, and elimination in both fasting and fed states were modeled. As the used software does not directly simulate intestinal lymphatic transport, lymphatic involvement in the fed state was examined indirectly through parameter adjustments such as first-pass metabolism, pKa-driven solubility changes, and bile-salt-mediated solubilization, with the aid of molecular dynamics simulations under post-prandial pH. **Results**: The pharmacokinetic models revealed a reduction in the first-pass effect of halofantrine in the fed state compared to that in the fasting state. While adjustments in metabolism kinetics sufficed for constructing a representative PBPK model in the fasting state, capturing the fed-state profile required both modifications to metabolism kinetics and other parameters related to the structural rearrangements of halofantrine driven by the changes in intestinal pH following food intake. These changes were confirmed using molecular dynamics simulations of halofantrine in pHs reflecting the post-prandial conditions. **Conclusions**: This study underscores the need for further exploration and direct modeling of intestinal lymphatic uptake via PBPK models, highlighting its underexplored status in simulation algorithms. Moreover, the importance of integrating representative physicochemical factors for drugs, particularly in post-prandial conditions or lipid formulations, is evident. Overall, these findings contribute to advancing predictive regulatory and developmental considerations in drug development using post hoc analyses.

## 1. Introduction

After absorption, dietary fats and lipophilic vitamins traverse intestinal enterocytes and are packaged into excretory enterocyte lipoproteins, known as chylomicrons. These chylomicrons primarily transport the absorbed fats and lipophilic xenobiotics into the bloodstream via the lymphatic network [1,2,3]. Particularly for drug molecules, especially lipophilic ones (with a log *p* > 5), hitchhiking on chylomicrons enables them to bypass first-pass liver metabolism, thus potentially enhancing their bioavailability through intestinal lymphatic absorption. Additionally, these extracellular lipoprotein particles show great promise for drugs targeting the lymphatic system, offering potential for enhancing therapeutic efficacy while minimizing off-target toxicity [1,3,4,5,6]. For several drugs, the intestinal lymphatic route has been used to enhance oral bioavailability and therapeutic activity. One example is that of testosterone, where lipophilic prodrug testosterone undecanoate facilitates lymphatic delivery and hence circumvents extensive first-pass metabolism [7,8]. Similar benefits in absorption and therapeutic outcomes have also been reported for other drugs, with numerous examples listed in previous reviews [1,9].

Despite the significance and unique characteristics of the intestinal lymphatic absorption pathway, the field of in silico modeling for lymphatic oral absorption remains relatively underexplored and underdeveloped [4,10]. To our knowledge, only one model has been reported, aiming to establish a quantitative relationship between molecular structure and the lymphatic transfer of lipophilic compounds co-administered with a long-chain triglyceride vehicle. Molecular descriptors were computed using the VolSurf computer program, and a structure–property relationship was established through a partial least-squares analysis (PLS). The predictive power of the PLS model was found to surpass that of the frequently used method correlating log *p* values (LogKow) with intestinal lymphatic transfer [11].

In the context of mechanistic in silico modeling, physiological-based pharmacokinetic (PBPK) modeling has emerged as a promising approach to assess drug exposure in virtual populations and to obtain mechanistic insight into drug characteristics [12,13,14,15]. PBPK models provide simulated concentration versus time profiles of a drug and its metabolite(s) in plasma or an organ of interest and simultaneously allow for estimation of a variety of PK parameters [16]. This modeling approach provides a mechanistic understanding of drug behavior by integrating physiological parameters, allowing for personalized predictions of drug pharmacokinetics and the assessment of interindividual variability [17]. It enables the prediction of first in-human dose selection and optimization of dosing regimens [18,19], drug interactions involving enzymes, transporters, and multiple interaction mechanisms [20,21], evaluation of drug disposition and safety in special populations [22], and other applications [15,16,20]. This approach is both commonly and increasingly being accepted by regulatory agencies as a valuable tool in investigational new drug (IND) development and new drug applications (NDAs) and for new formulation approvals [12,23].

Intestinal lymphatic uptake still remains a relatively overlooked aspect in drug development and clinical pharmacokinetics using PBPK modeling, despite the significant implications for drug absorption, especially with the growing use of lipid-based formulations such as solid-lipid nanoparticles and other formulations aimed at enhancing systemic absorption via this alternative pathway. Only one study was found utilizing the Simcyp software (V19 Release 1, Simcyp, Sheffield, UK), implementing a PBPK model incorporating intestinal lymphatic uptake through the Multi-layer Advanced Dissolution, Absorption, and Metabolism (M-ADAM) framework [24]. This work provided a foundation for integrating lymphatic transport into PBPK modeling. However, further refinement is needed to enhance the mechanistic representation of this pathway. The current study contributes to the growing body of knowledge on intestinal lymphatic uptake by employing a different modeling strategy, utilizing GastroPlus™ to systematically incorporate lymphatic uptake while distinguishing between fasting and fed-state absorption.

Halofantrine is a synthetic antimalarial agent with activity against chloroquine-resistant *P. falciparum* and is widely available outside the United States [25]. It demonstrates unique absorption characteristics, related to its erratic absorption [26]. To ensure consistent absorption and improved bioavailability of halofantrine, it is recommended to be administered with a fatty meal, leveraging its lipophilic nature to facilitate additional absorption through the intestinal lymphatics [27,28]. Modeling this process is essential for a more precise characterization of halofantrine’s pharmacokinetic profile, as well as its efficacy and safety. This study aims to refine PBPK modeling approaches by capturing the mechanistic impact of lymphatic transport, providing deeper insights into drug pharmacokinetics under different physiological conditions.

## 2. Materials and Methods

### 2.1. PK and PBPK Modeling of Halofantrine

The input parameters used in the pharmacokinetic and the physiologically-based pharmacokinetic models (PK and PBPK, respectively) were obtained via ADMET predictor 10.4 (AP 10.4, Simulations Plus, Lancaster, CA, USA) or from published and reported in vitro values. All input model parameters are listed in Table 1. The data for absorption, distribution, metabolism, and elimination of halofantrine in both fasted and fed states have been previously reported by Miton et al. [29], and were modeled and constructed using GastroPlus™ 9.8.3 (Simulation Plus, Inc., Lancaster, CA, USA). The Advanced Compartmental Absorption and Transit (ACAT™) model and PBPK Plus™ was utilized, alongside the drug metabolism module. Halofantrine pharmacokinetic parameters were determined utilizing the PKPlus tool within GastroPlus™ software. For the PBPK modeling, human organ weights, volume, and blood perfusion rates were derived from the Population Estimate of Age-Related (PEAR™) physiology module within GastroPlus™. Tissue plasma partition coefficients (Kp) were predicted using the default in silico Lukacova Kp method [30]. Metabolic clearance was estimated from in vitro K_m_ and V_max_ values of CYP3A4 sourced from published literature [31]. The primary pharmacokinetic parameters for the halofantrine oral profiles in the developed PBPK model (the area under the concentration–time curve from time zero to time t [AUC_0–t_], maximum plasma concentration (C_max_), and the time to reach that concentration (T_max_)) were assessed using fold error values (FE) and percentage prediction errors (%PE) (Equations (1) and (2), respectively).

(1) $$\mathrm{FE}=\;\;\frac{Predicted\;Mean\;Value}{Observed\;Mean\;Value}$$

(2) $$\%\mathrm{PE}=\;\;\frac{Predicted-Observed}{Observed}\times 100\%$$

The PK model was derived from a selected IV profile construct using data in Krishna et al. [37], where a 1 mg/kg IV infusion of halofantrine was administered over one hour to nine adults (primarily male). These individuals had a mean age of 21 years and an average weight of 52 kg.

The oral halofantrine pharmacokinetic profiles in both fasting and fed states were reported by Milton et al. [29]. In their study, six healthy males were administered a 250 mg tablet of halofantrine hydrochloride after an overnight fast. These same subjects underwent a washout period of at least 6 weeks before receiving the same dose in the same tablet form, after consuming a standardized fatty meal consisting of 60 g of fat (including sausage, scrambled egg, fried potato, and one pint, 568 mL, of whole pasteurized milk). The pharmacokinetic profiles obtained included mean values reported for all individuals with an average weight of 68 kg and a mean age of 28 years in both the fasting and fed states.

### 2.2. Parameter Sensitivity Analysis (PSA)

The systemic exposure of halofantrine was assessed under varying pKa conditions to evaluate parameter sensitivity. Bile salt solubilization ratio was taken as a surrogate for the pKa-dependent solubility shifts. At the baseline pKa of 8.16, the bile salt solubilization ratio was 2,280,000. When the pKa was lowered to 5.58, the ratio increased 16-fold to 36,200,000. In contrast, a reduced solubilization ratio of 142,500, representing a 16-fold decrease, was also evaluated relative to the baseline pKa of 8.16.

### 2.3. Molecular Dynamics (MD) Simulations of Halofantrine

**Figure 1:**
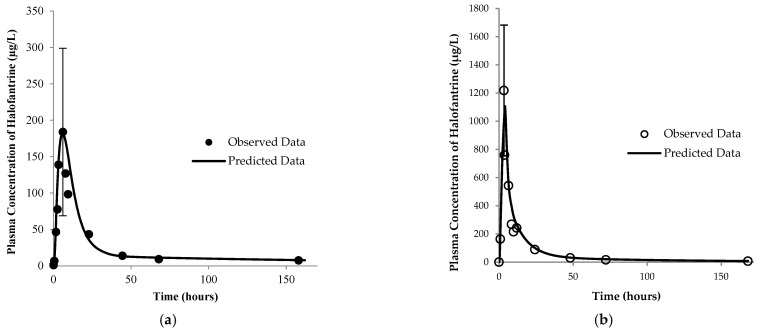
Pharmacokinetic (PK)-model-simulated and -observed (25) profiles of halofantrine in the (**a**) fasting and (**b**) fed states. Observed data represent the mean values for 6 subjects and the error bars represent the standard deviation of the observed highest plasma concentration (C_max_). Schemes follow another format.

The 3D conformer of halofantrine was obtained in the Structured Data File (SDF) format from the PubChem database (https://pubchem.ncbi.nlm.nih.gov). Protonated and unprotonated states at different pH levels (6.23 and 8.78) consistent with the values reported in [36], as well as their lowest-energy configurations, were generated using the structure preparation panel in the Molecular Operating Environment (MOE) [38]. The two ligand states were then prepared for molecular dynamics (MD) simulations using the ANTECHAMBER and tleap modules in AMBER20 [39]. Ligand parameters were assigned using the AM1-BCC charge method and the GAFF2 force field, and tleap was used to create parameters of the solvated complex [40,41]. In tleap, each system was solvated using the TIP3P aqueous model, extended 12 Å in all directions from the compound, and neutralized to a physiological ionic concentration of 0.15 M by adding 13 Na^+^ and Cl^−^ ions as shown in Figure 1. Molecular dynamics simulations were performed using the pmemd.cuda engine in AMBER20, utilizing GPU acceleration on the Graham cluster at Compute Canada. Each system underwent a standard molecular dynamics protocol that included energy minimization, heating, equilibration, and a 1000 ns production run with a 2 fs time. During the minimization process, conformational constraints with 50 kcal/mol were applied to the ligand atoms. During the heating phase, the temperature was gradually increased from 0 K to 310 K. The constraints were progressively removed during equilibration, which was performed at a constant temperature. The production phase involved an unconstrained 1000 ns MD simulation. A detailed molecular dynamics simulation protocol is available in reference [42]. The final trajectories were used to extract the dominant ligand configurations and analyze ligand water interactions using cpptraj and Visual Molecular Dynamics (VMD) [43,44].

## 3. Results

### 3.1. PK Modeling of Halofantrine

Several differences were noted in the halofantrine pharmacokinetic profiles in both the fasting and fed states (Figure 1). One important distinction between the two states was the decreased pre-systemic loss due to the first-pass effect in the fed state (9.9%) compared to the fasting state (77.2%). This is demonstrated through the absorption of halofantrine, as indicated by the maximum plasma concentration (C_max_) and an area under the curve up to time (t) (AUC_0–t_) (34) being higher in the fed state (Table 2).

### 3.2. PBPK Modeling of Halofantrine

**Figure 2:**
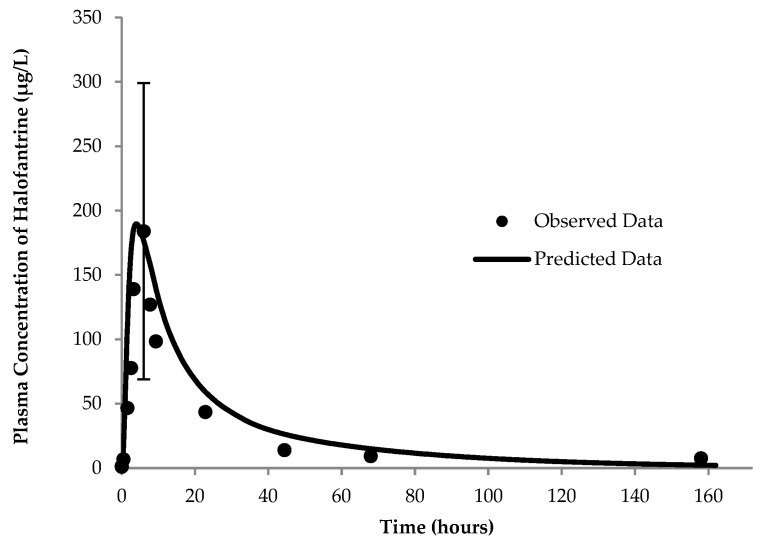
Physiologically based pharmacokinetic (PBPK)-model-simulated and -observed (25) profiles of halofantrine in the fasting state. Observed data represent the mean values for 6 subjects and the error bars represent the standard deviation of the observed highest plasma concentration (C_max_).

**Figure 3:**
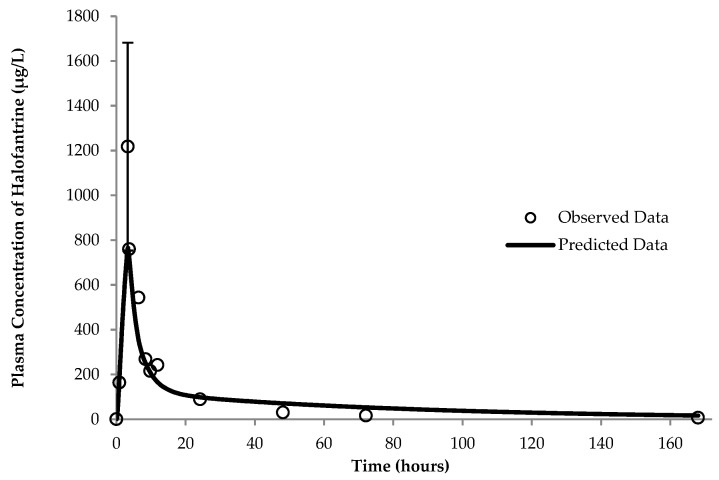
Physiologically based pharmacokinetic (PBPK)-model-simulated and -observed (25) profiles of halofantrine in the fed state. Observed data represent the mean values for 6 subjects and the error bars represent the standard deviation of the observed highest plasma concentration (C_max_).

Two PBPK models were developed for halofantrine for the fasting and fed states, respectively. The fasting state profile closely matched the reported data in terms of C_max_ and AUC_0–t_ for the drug, as illustrated in Figure 2 and summarized in Table 3. For the fed state, the final profile is depicted in Figure 3. Values of the different parameters in comparison to the observed data are in Table 3.

### 3.3. Parameter Sensitivity Analysis (PSA)

**Figure 4:**
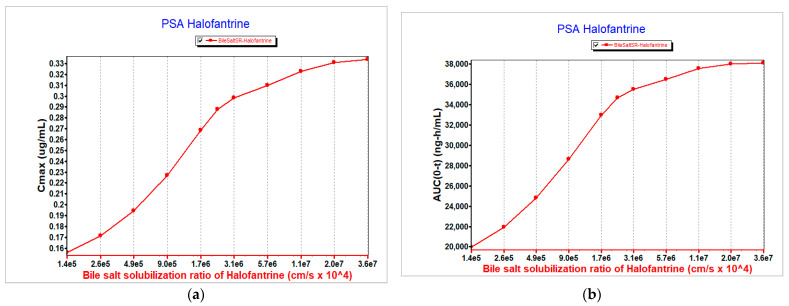
Effect of bile salt solubilization ratio on halofantrine exposure as indicated by (**a**) the maximum concentration reaching the systemic circulation (C_max_) and (**b**) area under the concentration–time curve (AUC_0–t_).

PSA supported that modifications in the solubilization ratio as a result of pKa shift influenced the systemic exposure of halofantrine as reflected by corresponding changes in its C_max_ and AUC_0–t_ (Figure 4).

### 3.4. MD Simulations of Halofantrine

To confirm these previously reported findings, molecular dynamics simulations were run to investigate the conformational behavior of halofantrine. Both the protonated and unprotonated states were examined for comparison, with complete simulation details presented in Supplementary File S3. One of the points of interest was the distance between the nitrogen atom and the hydroxyl oxygen, tracked over a 1000 ns MD simulation as a measure of hydrogen bond formation.

**Figure 5:**
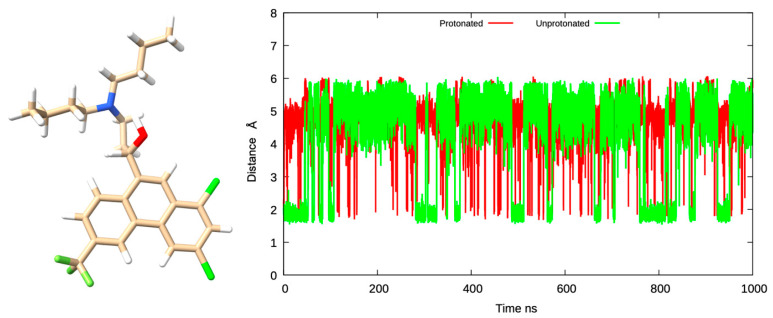
Monitoring the distance between nitrogen (blue) and oxygen (red) atoms during 1000 ns molecular dynamics simulations.

For the protonated state, the average distance was maintained at ~5 Å, with sporadic drops to ~2 Å, indicating negligible or transient interactions. The unprotonated state, however, displayed a more dynamic profile, with the distance fluctuating between 1.9 Å and 5 Å roughly every 25 ns. The frequent switching is indicative of the periodic formation and breaking of a hydrogen bond, which is suggestive of stronger and more persistent intramolecular interactions in the unprotonated form. These findings are depicted in Figure 5.

Consistent with this, MD trajectory analysis revealed the formation of a direct hydrogen bond between a protonated nitrogen atom and a hydroxyl oxygen, with an average donor–acceptor distance of approximately 3.5 Å. This interaction was observed in 4,537 frames, corresponding to occupancy of ~4.5%, indicating a transient yet recurring hydrogen bond. In contrast, in the unprotonated system, a hydrogen bond between a nitrogen atom and a hydroxyl group was detected in approximately 37.3% of the simulation (37,467 frames), with an average donor–acceptor distance of 2.74 Å, further supporting the stronger and more persistent nature of hydrogen bonding in this state.

## 4. Discussion

Gastroplus™ uses an advanced compartmental and transit (ACAT) model to predict absorption, distribution, metabolism, and excretion properties of drugs. In this model, the gastrointestinal tract is divided into nine compartments from the stomach to the ascending colon and accounts for the different physicochemical factors of the drug, along with the mechanistic descriptions of the underlying biophysical and biochemical processes to predict the intestinal drug absorption and in vivo drug behavior [35,36,37].

This work aimed at using Gastroplus™ to develop a PBPK model to investigate the behavior of halofantrine after oral administration in the fasting and the fed states, where in the latter, significant contribution of intestinal lymphatic uptake is likely enhanced.

For PBPK generation, determining the pharmacokinetic parameters through appropriate PK models is the first step to providing insights into the observed differences in drug plasma profiles in the fed and fasted states [29], which can be used to further develop and define the PBPK model and to help direct the building of a PBPK model based on the determined pharmacokinetic parameters.

### 4.1. PK Modeling of Halofantrine

To obtain an accurate and descriptive PK model, an intravenous (IV) dose profile is essential to utilize in conjunction with the oral plasma profile. Intravenous administration usually provides complete bioavailability and drug delivery into the bloodstream, allowing the isolation of the absorption phase, which can be used to establish a baseline pharmacokinetic profile and model pharmacokinetic parameters such as total body clearance, volume of distribution, and elimination of half-life without the possibility of contamination by unabsorbed drugs [45]. Additionally, it will act as a reference when calculating the oral bioavailability by comparing the oral profile to the intravenous profile from Krishna et al. [37,46,47].

For the halofantrine profiles in both states (fasting and fed), identical input parameters for the drug were utilized, as outlined in Table 1. However, the fasting-state physiology of the human body was employed for modeling the fasting-state profile, while the fed state (characterized by high fat and high calorie intake) was chosen for the fed-state profile. Additionally, the fed-state profile incorporated a shift to zero-order gastric emptying kinetics where the gastric emptying time is calculated based on a linear correlation to the caloric content of the meal. Moreover, the bile salt concentrations in the intestinal compartments are calculated based on the percentage of fat in the meal [48].

Since halofantrine undergoes extensive metabolism in the liver [27], absorption via the lymphatic pathway can provide an advantage by allowing a portion of the drug to bypass hepatic first-pass metabolism. Appreciably, a greater extent of halofantrine evaded the first-pass extraction by the liver when administered in the modeled fed state. This enhanced absorption of halofantrine could be facilitated by the absorption through the intestinal lymphatic pathway, which anatomically bypasses the liver, reducing first-pass effects, and leads to higher systemic exposure of the drug [28,49].

The theoretical effect of food on the extraction ratio of a high-extraction-ratio (ER) drug suggests that in the fed state, the reduced first-pass effect results in a higher proportion of the drug reaching systemic circulation compared to the fasting state, and a lower extraction ratio would be evident. This phenomenon where a high-ER drug in fasted conditions is changed to a lower-ER drug under fed conditions can lead to an increased C_max,_ and AUC, promoting enhanced oral drug bioavailability (F) in the fed state due to reduced hepatic extraction and thus more drug available for lymphatic absorption. An increase in drug AUC with increases in the fraction absorbed lymphatically is highly pronounced with a high-ER drug (>10-fold), along with significant increases in C_max_ and AUC [50].

Another consideration noticed between the halofantrine fasting- and fed-state profiles is the number of compartments necessary to accurately model each profile (Supplementary Files S1 and S2, respectively). While a two-compartment model adequately represented the fasting-state condition, applying the two-compartment model to the fed-state profile failed to precisely capture its pharmacokinetic profile, as evidenced by two crucial goodness-of-fit criteria—the Akaike Information Criterion (AIC) and Schwarz Criterion (SC) [51,52]. For the fed-state profile, the AIC and SC values for the two-compartment model were −24.954 and −17.643, respectively. In contrast, the three-compartment model exhibited a markedly improved fit, with an AIC of −60.348 and an SC value of −50.947, both more than two-fold lower. Therefore, the three-compartment model was selected. As noted in Table 2, for the developed PK models in both states, the predicted PK parameter values were within the acceptable limit of prediction (< 1.3-fold error [53] and %PE ≤ 50% [54]).

**Figure 6:**
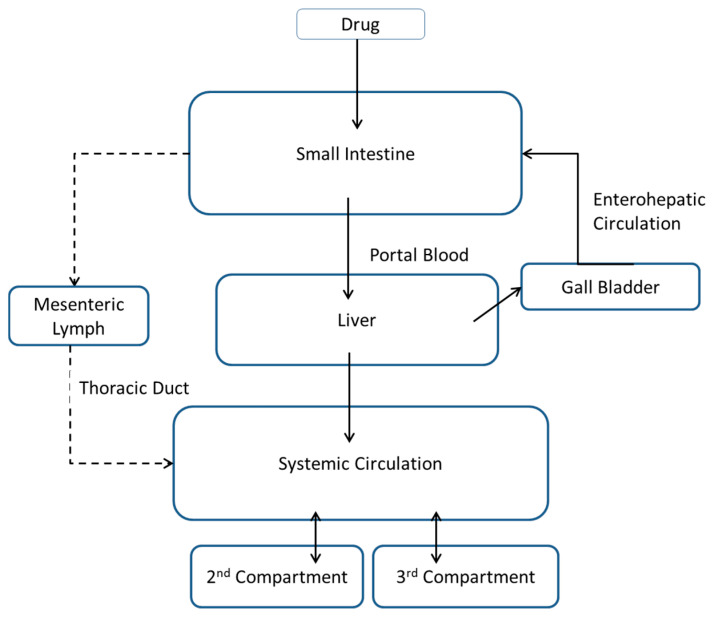
Diagram depicts the absorption pathway adopted in the in silico models for orally administered drugs, utilizing continuous arrows to signify the route from the intestine through the portal vein to the liver and subsequently to the general circulation. Additionally, the overlooked lymphatic absorption pathway is illustrated with dotted arrows, indicating that drugs can travel through the mesenteric lymph to enter the general circulation.

In pharmacokinetic modeling, a two-compartment model typically represents drug distribution between the central compartment (blood) and a peripheral compartment (tissue). This model assumes that drug distribution throughout the body can be adequately described by these two compartments. However, in some cases, particularly when considering complex absorption processes or additional tissue compartments, a two-compartment model may not fully capture the behavior of drugs. In cases where there are significant delays in absorption, extensive tissue distribution, multiple metabolic pathways, lymphatic or lysosomal trapping, in addition to other scenarios, a more comprehensive approach with additional compartments may offer a more comprehensive understanding of the pharmacokinetic behavior of drugs (Figure 6). The decision to use a multi-compartment model depends on the specific characteristics of the drug being studied and the objectives of the analysis. However, introducing a third compartment in this scenario enables a more nuanced representation of the distribution and elimination kinetics of the drug under different conditions [55,56].

In the case of the fed-state profile of halofantrine, the addition of a third compartment was proposed to better describe its disposition characteristics when taken with a fatty meal. Compartmental PK modeling in this software (PKPlus 2.0) is built so that the drug, once crossing the basolateral intestinal barrier, proceeds to the portal vein and subsequently enters the liver. Upon evading first-pass metabolism, the drug gains access to the systemic circulation, allowing distribution to peripheral compartments (Figure 6). Thus, the third compartment can be related theoretically to the undetermined mechanistic distribution or the uptake through the lymphatics which is involved and enhanced in its absorption postprandially. Yet, the lymphatic absorption pathway is not incorporated directly into the software but accounted for indirectly in the ACAT model with the bile salt concentrations after a fatty meal and the solubility of the drug in the FeSSIF condition. Therefore, the third compartment was linked to a process that would be captured by the software building, and it is related to the disposition of the drug after reaching the general circulation which is the lysosomal trapping. Halofantrine, being a weak basic lipophilic drug, is reported to be trapped inside cellular lysosomes (lysosomotropic) [57,58]. This additional tissue compartment accounts for the third compartment within the fed-state profile.

Capturing lysosomal trapping in the fed-state profile rather than the fasting-state profile may be attributed to the higher drug concentration reaching the general circulation and tissues, including lysosomes, when in the fed state compared to in the fasting state. In the fasting state, the goodness of fit of the three-compartment model exhibited significant decline compared to the two-compartment model. Adjusting the unbound fraction in the enterocyte (F_uent_) in the fasting state facilitated the lysosomally trapped portion of halofantrine, leading to the development of a more representative PK model.

Although the built pharmacokinetic models were able to describe the plasma concentration–time profile of halofantrine in humans, they did not capture the mechanistic contribution of lymphatic uptake. There are no direct parameters such as drug lymphatic concentrations or the proportion of absorbed dose carried via the lymph in humans because dynamic lymph measurements using techniques like mesenteric lymph duct cannulation are technically and ethically infeasible in clinical studies [9].

In this work, PK pharmacokinetic parameters derived from PK modeling were used as a basis to build a PBPK model in which lymphatic transport can be incorporated indirectly. Insights into this pathway are available from preclinical studies, particularly rat and dog experiments employing lymph duct cannulation [59,60]. Future studies could leverage these data to extrapolate lymphatic uptake parameters from preclinical animals to humans [61].

### 4.2. PBPK Modeling of Halofantrine

The genesis here is a PBPK model for the fasting-state profile of halofantrine using physicochemical properties derived from the values presented in Table 1 and the profiles documented by Milton et al. [29].

Given the low solubility and high permeability of halofantrine (biopharmaceutical classification system, BCS, Class II drug) [62], the distribution of the drug was determined to be perfusion-rate-limited. With the perfusion-limited distribution, the accumulation rate of the drug in tissues is constrained by the blood flow rate within the tissue (perfusion rate) [63]. Instantaneous partitioning is assumed, where the tissue’s specific partition coefficient (Kp) is employed to determine drug partitioning between plasma and the entire tissue at each time interval. As a result, the drug concentration in the tissue is calculated as the product of the tissue Kp and the instantaneous drug concentration in the plasma during the relevant time period [64].

Since halofantrine is a weakly basic drug (Table 1), it would remain ionized at urine pH, making active tubular secretion unlikely [65]. Additionally, neither active secretion nor reabsorption has been reported in the literature. Consequently, renal elimination is expected to be primarily governed by glomerular filtration of the unbound drug. Renal clearance was set to (glomerular filtration rate * unbound drug fraction (GFR * F_u_)) to account for the portion cleared through the kidneys. Halofantrine is primarily metabolized by the liver, chiefly through CYP_3A4_ [66,67]; thus, the K_m_ and V_max_ of this enzyme were incorporated, based on the literature data [31].

The initial profile of the fasting state, shaped by these inputs, aligned with the observed data but indicated a greater clearance phase than observed. Optimization of the metabolism constant and maximum velocity of enzyme reaction (K_m_ and V_max_, respectively) values for CYP_3A4_ resulted in the profile presented in Figure 2. The model predictions yielded a pharmacokinetic profile with parameters (Table 3) falling within the acceptable prediction limits as defined earlier [53,54].

In a physiologically based model, changing the physiology to the fed-state would often predict the fed-state plasma profile especially for low-extraction-ratio drugs [68,69]. Thus, after uploading the observed fed-state profile, and adjusting the physiological conditions to reflect the fed state (characterized by high fat and high calories), the zero-order emptying option was selected for as previously mentioned. However, in this scenario, a change in the fed-state physiology is involved, but an additional absorption route with pharmacokinetic implication related to the first-pass effect is suggested. As the intestinal lymphatic uptake cannot yet be directly simulated here, then reducing the first-pass percentage as shown with the PK modeling would be a suitable potential approach to adjust the PBPK model in the fed state to reflect the increase in the drug absorption caused by reduced hepatic extraction and intestinal lymphatic uptake. Firstly, the linear clearance obtained from the non-compartment model of the concentration–time profile used was employed to develop the model in the fed state. However, the observed C_max_ value was 2.6 times higher than the predicted one.

**Figure 7:**
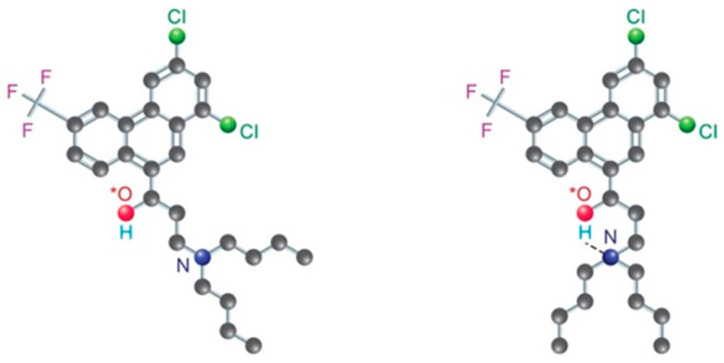
Illustration showing the side chain folding, drawing the tertiary amine nearer to the benzylic hydroxyl group (*). This spatial configuration creates an intramolecular hydrogen bond, consequently lowering the basicity of amine in halofantrine and changing the pKa from 8.16 for the left molecular configuration to 5.58 for the right molecular configuration.

Halofantrine, being a tertiary aliphatic amine, is expected to have a high pKa. The calculated pKa was found to be 8.16 [32] as used in all previously described models. However, in a representative fed-state medium (pH = 6), it has been documented in the literature that halofantrine has a pKa of 5.58 ± 0.07 [33]. Molecular modeling calculations have suggested that the decrease in pKa might result from the folding of the side chain, bringing the tertiary amine closer to the benzylic hydroxyl group. This spatial arrangement forms an intramolecular hydrogen bond, reducing the basicity of the amine in halofantrine (Figure 7) [33].

Including this updated pKa (5.58) in the fed-state PBPK model, in alignment with the underlying molecular dynamics, the solubilization was enhanced by 16 times and a plasma concentration–time profile was provided that was very similar to the observed clinical data and within statistically acceptable limits [53,54] (Figure 3 and Table 3).

From a physiological perspective, the lower pKa would enhance the affinity for products of lipid digestion, facilitating its absorption which was predicted to occur primarily (80%) in the duodenum and jejunum (pH = 5.4–6, according to the ACAT Model, supplementary). These segments have a higher density of villi [70], where lymphatic capillaries (lacteals) are located [2] which will facilitate the traverse of halofantrine through the intestinal pathway into the general circulation, bypassing the first-pass liver extraction and clearance, leading to increased fraction absorbed.

Both first-pass evasion and enhanced solubilization played roles in modeling the lymphatic uptake of halofantrine in the fed state. While first-pass metabolism adjustments alone did not sufficiently capture the observed pharmacokinetics, integrating the increased solubility under fed conditions improved capturing the observed pharmacokinetics. Furthermore, analysis of the data from Milton et al. suggests that the post-C_max_ profiles are parallel in both conditions [29], indicating no difference in half-life. This implies that the increased exposure in the fed state is primarily due to improved bioavailability (F). The enhanced solubilization facilitated a greater fraction absorbed (F_a_) in the gastrointestinal tract likely through the intestinal route as reported in the literature [28,33], contributing to a higher bioavailable dose. Since bioavailability (F) is a product of F_a_, the fraction escaping intestinal first-pass metabolism (F_g_), and the fraction escaping hepatic first-pass metabolism (F_h_) (i.e., F = F_a_ * F_g_ * F_h_), both an increase in F_a_ through the intestinal lymphatics and a reduction in first-pass extraction as a result of the lymphatic voyage could contribute to the improved systemic availability of halofantrine [9,71].

It is also important to acknowledge the potential for overestimation of lymphatic contribution using this indirect modeling technique. As discussed in the previous section (Section 4.1), there are no direct lymphatic human measurements. A potential step towards external validation would be to compare simulated outputs with the measurement of lymph duct-cannulated animal models [59,60] that provide quantitative measurements of drug concentration in mesenteric lymph.

As demonstrated, developing a novel PBPK model to reflect the intestinal lymphatic uptake is not a straightforward process, at least using the existing algorithms in Gastroplus^TM^. For high-E drugs, the most prominent and likely effect would be related to changes in pre-systemic metabolism; then, adjusting the enzyme kinetics might help reflect additional intestinal lymphatic uptake. However, for medium- and low-ER drugs, where adjusting the metabolism profile might not be as efficient in reflecting drugs’ lymphatic uptake, empirical adjustment of the different properties of individual gastrointestinal compartments might also be an option. Important considerations from this could be the significance of updating the ACAT model to accommodate the intestinal lymphatic uptake route to further characterize the model and dual absorption processes of drugs reaching the general circulation through intestinal lymphatics. For this, adopting M-ADAM-like frameworks included in platforms such as Simcyp may enable direct representation of lymphatic partitioning [24]. Additionally, integrating the relevant equations and functions is crucial to accurately address the impact of this route on the metabolism and disposition of potential drugs and the increasingly prevalent lipid formulations utilized for enhancing hydrophobic drug delivery. Moreover, future strategies may involve more explicit mechanistic representations of lymphatic transport by including drug–chylomicron interaction simulations which can be an effective indicator of lymphatic uptake, with a more direct link to the physiology. Also, scoring of the different molecular descriptors favoring or disfavoring the drug–chylomicron association can serve the same purpose [72]. By including such updates in the model, pharmaceutical scientists could more optimally simulate the absorption kinetics of lymphotropics and rationally account for the impact of food or formulation on their pharmacokinetics.

## 5. Conclusions

This study presents an initial investigation into the in silico modeling of intestinal lymphatic uptake using halofantrine as a case study. Theoretical considerations regarding the impact of food on the absorption of lymphotropics like halofantrine offer avenues to modulate PBPK models for such drugs when administered in formulations targeting lymphatics or alongside food effects that can enhance intestinal lymphatic uptake. Nevertheless, for accurate pharmacokinetic predictions, experimental and mechanistic information needs to be incorporated into modeling strategies. In this work, the effect of food on halofantrine absorption was linked to a change in its pKa under post-prandial intestinal conditions. Such food-induced molecular changes were instrumental in an accurate description of halofantrine behavior in the fed-state PBPK model. Additionally, alternative measures may be considered for other drugs. Nonetheless, all these methods are indirect, underscoring the necessity for developing alternative and direct modeling options for this critical absorption route. Moving forward, a MADAM-like framework may be applied to represent lymphatic uptake directly. Also, integrating drug–chylomicron interaction kinetics into currently available software, together with molecular descriptors indicating lymphatic voyage, may assist in simulating lymphotropic drug absorption via the chylomicron pathway, supporting translational pharmacokinetic modeling and drug development strategies.

## Figures and Tables

**Table 1:** Input parameters for the different properties of halofantrine used in developed PK and PBPK models.

Property	Value	Reference
Molecular weight (g/mol)	500.44	AP 10.4
pKa	8.18	[32]
pKa	5.58	[33]
Log *p*	7.58	AP 10.4
Aqueous solubility (mg/mL)	0.0001 (pH = 1.2) 0.00024 (pH =7.4)	[34]
FaSSIF (mg/mL)	0.00552 (pH = 6)	[33]
FeSSIF (mg/mL)	2.311 (pH = 6)	[33]
Solubility factor	105,000	AP 10.4
Permeability (cm/s 10E^4^)	1.44	AP 10.4
Percent unbound in plasma (%)	0.7 *	[35]
Blood/plasma concentration ratio	1.5	[35,36]
In vitro CYP3A4 K_m_ halofantrine (µM)	48	[31]
In vitro CYP3A4 V_max_ halofantrine (pmol min^−1^ mg^−1^ protein)	215	[31]

* F_uent_ (fraction unbound in enterocyte) = 2% to account for lysosomal trapping.

**Table 2:** PK model parameters for simulated and observed [29] fasting and fed states of halofantrine, with corresponding fold error values (FE) and percentage prediction errors (%PE).

PK Parameter	Observed Value ± s.d. (*n* = 6)	Predicted Value	FE	%PE
Fasting State				
C_max_ (µg/L)	184.0 ± 115.0	181.5	0.98	1.4
T_max_ (h)	6.0 ± 1.3	5.8	0.97	3.3
AUC_0–t_ (µg·L^−1^·h)	3.9 ± 2.6	3.9	1	0.0
Fed State				
C_max_ (µg/L)	1218.0 ± 464.0	1106.6	0.91	9.1
T_max_ (h)	3.3 ± 1.5	4.1	1.24	24.2
AUC_0–t_ (µg·L^−1^·h)	11.3 ± 3.5	11.9	1.05	5.3

s.d. represents the standard deviation.

**Table 3:** PBPK model parameters for simulated and observed [29] fasting and fed states of halofantrine, and the associated fold error values (FE) and percentage prediction errors (%PE).

PK Parameter	Observed Value ± s.d. (*n* = 6)	Predicted Value	FE	%PE
Fasting State				
C_max_ (µg/L)	184.0 ± 115.0	189.7	1.03	3.1
T_max_ (h)	6.0 ± 1.3	3.9	0.65	35
AUC_0–t_ (µg·L^−1^·h)	3.9 ± 2.6	4.5	1.15	15.4
Fed State				
C_max_ (µg/L)	1218.0 ± 464.0	764.9	0.63	37.2
T_max_ (h)	3.3 ± 1.5	3.3	1	0
AUC_0–t_ (µg·L^−1^·h)	11.3 ± 3.5	12.3	1.09	8.8

s.d. represents the standard deviation.

## Data Availability

All data is available from authors.

## Supplementary Materials

The following supporting information can be downloaded at: https://www.mdpi.com/article/10.3390/pharmaceutics17091228/s1, Supplementary File S1: GastroPlus^®^ 9.8.3 PK Input Data, Non-Compartmental, and Compart-mental Analysis (NCA) Results for Halofantrine Fasted State; Supplementary File S2: GastroPlus^®^ 9.8.3 PK Input Data, Non-Compartmental, and Compartmental Analysis (NCA) Results for Halofantrine Fed State; Supplementary File S3: Molecular Dynamics (MD) Simulations of Halo-fantrine.
