# Supplementary material for: Indirect Modeling of Post-Prandial Intestinal Lymphatic Uptake of Halofantrine Using PBPK Approaches: Limitations and Implications
Source: Pharmaceutics. 2025 Sep 22;17(9):1228. doi: 10.3390/pharmaceutics17091228 (PMC12473779; doi:10.3390/pharmaceutics17091228)
Supplement: Supplementary file 1 [file pharmaceutics-17-01228-s001.zip › Supplementary Files S1 .pdf]

# Modeling Intestinal Lymphatic Uptake of Halofantrine Post-prandially: Advancements and Implications in Physiologically Based Pharmacokinetic Modeling

**Supplementary Files S1**

GastroPlus® 9.8.3 PK Input Data, Non-Compartmental, and Compartmental Analysis (NCA) Results for Halofantrine Fasted State

ORAL CP-TIME DATA FROM FILE:

| Time, h | C, ng/mL |
|---|---|
| 0 | 1 |
| 0 | 0.981 |
| 0.027 | 1.54 |
| 0.449 | 6.77 |
| 1.55 | 46.7 |
| 2.55 | 77.7 |
| 3.23 | 139 |
| 5.51 | 187 |
| 7.74 | 127 |
| 9.34 | 98.4 |
| 22.8 | 43.5 |
| 44.4 | 13.9 |
| 67.9 | 9.28 |
| 158 | 7.58 |

DOSING INFORMATION:

Oral Dose Dose = 250 mg

NONCOMPARTMENTAL ANALYSIS OF DATA:

| | | |
|---|---|---|
| AUC(0-t) = | 3.676 | µg-h/mL |
| AUC(0-inf) = | 7.051 | µg-h/mL |
| AUMC = | 2169.2 | µg-h^2/mL |
| MRT = | 307.6 | h |
| CL/F = | 35.46 | L/h |
| K(z) = | 2.25E-03 | 1/h |
| t 1/2 = | 308.6 | h |
| Vss/F = | 1.09E+04 | L |

C(0) bolus = 0. µg/mL

IV CP-TIME DATA FROM FILE:

| Time, h | C, ng/mL |
|---|---|
| 0 | 3.72 |
| 0.518 | 740 |
| 1.07 | 933 |
| 1.54 | 255 |
| 2.05 | 184 |
| 3.04 | 143 |
| 4.01 | 109 |
| 5.95 | 93.1 |
| 8.02 | 73.4 |

DOSING INFORMATION:

IV Infusion
Dose= 52 mg Infusion time = 1 h

NONCOMPARTMENTAL ANALYSIS OF DATA:

| | | |
|---|---|---|
| AUC(0-t) = | 1.698 | µg-h/mL |
| AUC(0-inf) = | 2.337 | µg-h/mL |
| AUMC = | 14.66 | µg-h^2/mL |
| MRT = | 5.774 | h |
| CL = | 22.25 | L/h |
| K(z) = | 0.115 | 1/h |
| t 1/2 = | 6.035 | h |
| Vss = | 128.5 | L |

C(0) bolus = 0. µg/mL

==============================================

ONE-COMPARTMENT SIMULATION LINEAR MODEL:
================================================

| | | | |
|---|---|---|---|
| CL = | 1.088 | L/h | CV= 140.3% |
| Vd = | 54.91 | L | CV= 85.47% |
| CL/kg= | 0.018 | L/h/kg | CV= 140.3% |
| Vd/kg= | 0.915 | L/kg | CV= 85.47% |
| | | | |
| K10 = | 0.02 | 1/h | CV= 164.28% |
| | | | |
| Tlag = | 0.365 | h | CV= 172.74% |
| Ka = | 0.564 | 1/h | CV= 443.28% |
| F = | 4.161 | % | CV= 172.74% |
| | | | |
| Cmax = | 0.018 | ug/mL/mg Dose | |
| t 1/2 = | 34.99 | h | CV= 164.28% |
| | | | |
| AUC = | 2.337 | µg-h/mL | CV= 216.04% |
| AUMC = | 2437.8 | µg-h^2/mL | CV= 216.04% |
| MRT = | 50.49 | h | CV= 164.28% |

R^2 = -1.8476

Akaike Information Criterion (AIC) =
(#Pts) * Log(Obj) + 2(#Parameters)
= -0.0643

Schwarz Criterion (SC) =
(#Pts) * Log(Obj) + (#Parameters)*(Log(#Pts))
= 5.6131

Optimization time: 1.28125 sec
Total simulations: 1380

Weighted sum of squared errors = 6.456E-1
Weighting: 1/Yhat^2

------------------------------------------------------------------------
PREDICTED AND OBSERVED VALUES
---------------------------------------------------------------------------------------------

| Time | Pred | Obs | Residual |
|---|---|---|---|
| (h) | (ng/mL) | (ng/mL) | (ng/mL) |

| | | | |
|---|---|---|---|
| 0 | 0 | 1 | -1 |
| 0 | 0 | 0.981 | -0.981 |
| 0.027 | 0 | 1.54 | -1.54 |
| 0.449 | 6.717 | 6.77 | -0.053 |
| 1.55 | 69.679 | 46.7 | 22.979 |
| 2.55 | 99.972 | 77.7 | 22.272 |
| 3.23 | 111.998 | 139 | -27.002 |
| 5.51 | 127.341 | 187 | -59.659 |
| 7.74 | 127.395 | 127 | 0.395 |
| 9.34 | 124.745 | 98.4 | 26.345 |
| 22.8 | 96.282 | 43.5 | 52.782 |
| 44.4 | 62.768 | 13.9 | 48.868 |
| 67.9 | 39.408 | 9.28 | 30.128 |
| 158 | 6.615 | 7.58 | -0.965 |
| 0 | 0 | 3.72 | -3.72 |
| 0.518 | 488.055 | 740 | -251.945 |
| 1.07 | 936.436 | 933 | 3.436 |
| 1.54 | 927.759 | 255 | 672.759 |
| 2.05 | 918.434 | 184 | 734.434 |
| 3.04 | 900.6 | 143 | 757.6 |
| 4.01 | 883.462 | 109 | 774.462 |
| 5.95 | 850.157 | 93.1 | 757.057 |
| 8.02 | 816.005 | 73.4 | 742.605 |

==================================================

TWO-COMPARTMENT SIMULATION LINEAR MODEL:

==================================================

| | | | |
|---|---|---|---|
| CL = | 8.09 | L/h | CV= 147.47% |
| Vc = | 46.96 | L | CV= 83.41% |
| CL2 = | 12.11 | L/h | CV= 106.56% |
| V2 = | 1205.5 | L | CV= 0.0% |
| CL/kg= | 0.135 | L/h/kg | CV= 147.47% |
| Vc/kg= | 0.783 | L/kg | CV= 83.41% |
| CL2/kg= | 0.202 | L/h/kg | CV= 106.56% |
| V2/kg= | 20.09 | L/kg | CV= 0.0% |
| | | | |
| K10 = | 0.172 | 1/h | CV= 169.42% |
| K12 = | 0.258 | 1/h | CV= 135.32% |
| K21 = | 0.01 | 1/h | CV= 106.56% |
| | | | |
| Tlag = | 0.3742 | h | CV= 113.48% |
| Ka = | 0.098 | 1/h | CV= 140.15% |
| F = | 22.84 | % | CV= 113.48% |
| | | | |
| Cmax = | 0.017 | ug/mL/mg Dose | |

t 1/2 = 174.7 h CV= 0.0%
C* = 1.107 μg/mL

R^2 = 0.5759

Akaike Information Criterion (AIC) =
(#Pts) * Log(Obj) + 2(#Parameters)
= -18.2966

Schwarz Criterion (SC) =
(#Pts) * Log(Obj) + (#Parameters)*(Log(#Pts))
= -10.3482

Optimization time: 4.070313 sec.
Total simulations: 5278

Weighted sum of squared errors = 2.4556E-1
Weighting: 1/Yhat^2

-----------------------------------------------------------------------------------------------
PREDICTED AND OBSERVED VALUES
-----------------------------------------------------------------------------------------------

| Time | Pred | Obs | Residual |
|---|---|---|---|
| (h) | (ng/mL) | (ng/mL) | (ng/mL) |
| 0 | 0 | 1 | -1 |
| 0 | 0 | 0.981 | -0.981 |
| 0.027 | 0 | 1.54 | -1.54 |
| 0.449 | 6.672 | 6.77 | -0.098 |
| 1.55 | 78.981 | 46.7 | 32.281 |
| 2.55 | 114.153 | 77.7 | 36.453 |
| 3.23 | 127.374 | 139 | -11.626 |
| 5.51 | 137.295 | 187 | -49.705 |
| 7.74 | 124.899 | 127 | -2.101 |
| 9.34 | 112.446 | 98.4 | 14.046 |
| 22.8 | 40.475 | 43.5 | -3.025 |
| 44.4 | 14.827 | 13.9 | 0.927 |
| 67.9 | 10.763 | 9.28 | 1.483 |
| 158 | 7.291 | 7.58 | -0.289 |
| 0 | 0 | 3.72 | -3.72 |
| 0.518 | 514.203 | 740 | -225.797 |
| 1.07 | 873.68 | 933 | -59.32 |
| 1.54 | 714.598 | 255 | 459.598 |
| 2.05 | 575.143 | 184 | 391.143 |
| 3.04 | 378.832 | 143 | 235.832 |

| | | | |
|---|---|---|---|
| 4.01 | 253.406 | 109 | 144.406 |
| 5.95 | 117.378 | 93.1 | 24.278 |
| 8.02 | 56.522 | 73.4 | -16.878 |

==================================================

THREE-COMPARTMENT SIMULATION LINEAR MODEL:

| | | | |
|---|---|---|---|
| CL = | 6.621 | L/h | CV= 91.06% |
| Vc = | 46.98 | L | CV= 83.6% |
| CL2 = | 12.05 | L/h | CV= 107.33% |
| V2 = | 1176.8 | L | CV= 0.0% |
| CL3 = | 1.555 | L/h | CV= 380.51% |
| V3= | 8.47E+04 | L | CV= 0.0% |
| CL/kg= | 0.11 | L/h/kg | CV= 91.06% |
| Vc/kg= | 0.783 | L/kg | CV= 83.6% |
| CL2/kg= | 0.201 | L/h/kg | CV= 107.33% |
| V2/kg= | 19.61 | L/kg | CV= 0.0% |
| CL3/kg= | 0.026 | L/h/kg | CV= 380.51% |
| V3/kg= | 1411.5 | L/kg | CV= 0.0% |
| | | | |
| K10 = | 0.141 | 1/h | CV= 123.61% |
| K12 = | 0.256 | 1/h | CV= 136.05% |
| K21 = | 0.01 | 1/h | CV= 107.33% |
| K13 = | 0.033 | 1/h | CV= 389.58% |
| K31 = | 1.84E-05 | 1/h | CV= 380.51% |
| | | | |
| Tlag= | 0.374 | h | CV= 113.82% |
| Ka= | 0.098 | 1/h | CV= 140.89% |
| F= | 22.83 | % | CV= 113.82% |
| Cmax = | 0.017 | ug/mL/mg Dose | |
| C* = | 1.107 | ug/mL | |
| t 1/2 = | 4.66E+04 | h | CV= 0.0% |
| R^2 = | 0.5769 | | |

Akaike Information Criterion (AIC) =
(#Pts) * Log(Obj) + 2(#Parameters)
= -14.2976

Schwarz Criterion (SC) =
(#Pts) * Log(Obj) + (#Parameters)*(Log(#Pts))
= -4.0781

Optimization time: 2.46875 sec.

Total simulations: 2838

Weighted sum of squared errors = 2.4555E-1
Weighting: 1/Yhat^

-----------------------------------------------------------------------------------------------

PREDICTED AND OBSERVED VALUES

-----------------------------------------------------------------------------------------------

| Time | Pred | Obs | Residual |
|---|---|---|---|
| (h) | (ng/mL) | (ng/mL) | (ng/mL) |
| 0 | 0 | 1 | -1 |
| 0 | 0 | 0.981 | -0.981 |
| 0.027 | 0 | 1.54 | -1.54 |
| 0.449 | 6.683 | 6.77 | -0.087 |
| 1.55 | 79.033 | 46.7 | 32.333 |
| 2.55 | 114.195 | 77.7 | 36.495 |
| 3.23 | 127.399 | 139 | -11.601 |
| 5.51 | 137.255 | 187 | -49.745 |
| 7.74 | 124.819 | 127 | -2.181 |
| 9.34 | 112.354 | 98.4 | 13.954 |
| 22.8 | 40.458 | 43.5 | -3.042 |
| 44.4 | 14.887 | 13.9 | 0.987 |
| 67.9 | 10.815 | 9.28 | 1.535 |
| 158 | 7.26 | 7.58 | -0.32 |
| 0 | 0 | 3.72 | -3.72 |
| 0.518 | 513.965 | 740 | -226.035 |
| 1.07 | 873.17 | 933 | -59.83 |
| 1.54 | 714.043 | 255 | 459.043 |
| 2.05 | 574.586 | 184 | 390.586 |
| 3.04 | 378.345 | 143 | 235.345 |
| 4.01 | 253.027 | 109 | 144.027 |
| 5.95 | 117.212 | 93.1 | 24.112 |
| 8.02 | 56.511 | 73.4 | -16.889 |

*********************************************************************

AIC indicates two-compartment model is preferred.
SC indicates two-compartment model is preferred.

*********************************************************************

Search Method: Hooke & Jeeves Pattern Search
Weighting: 1/Yhat^2
