# Supplementary material for: Indirect Modeling of Post-Prandial Intestinal Lymphatic Uptake of Halofantrine Using PBPK Approaches: Limitations and Implications
Source: Pharmaceutics. 2025 Sep 22;17(9):1228. doi: 10.3390/pharmaceutics17091228 (PMC12473779; doi:10.3390/pharmaceutics17091228)
Supplement: Supplementary file 1 [file pharmaceutics-17-01228-s001.zip › Supplementary Files S2.pdf]

Modeling Intestinal Lymphatic Uptake of Halofantrine Post-prandially: Advancements and Implications in Physiologically Based Pharmacokinetic Modeling

Supplementary Files S2

GastroPlus® 9.8.3 PK Input Data, Non-Compartmental, and Compartmental Analysis (NCA) Results for Halofantrine Fed State

ORAL CP-TIME DATA FROM FILE:

Time, h	C, ng/mL
0	1
0.875	164
1.97	1218
3.67	760
6.4	544
8.43	269
9.79	216
11.9	243
24.2	89.5
48.1	31
72.1	16.9
168	7.58

DOSING INFORMATION:

Oral Dose Dose = 250 mg

NONCOMPARTMENTAL ANALYSIS OF DATA:

AUC(0-t) =	11.16	µg-h/mL
AUC(0-inf) =	12.07	µg-h/mL
AUMC =	516.1	µg-h^2/mL
MRT =	42.76	h
CL/F =	20.71	L/h
K(z) =	8.36E-03	1/h
t 1/2 =	82.9	h
Vss/F =	885.7	L

C(0) bolus = 0. µg/mL

IV CP-TIME DATA FROM FILE:

Time, h	C, ng/mL
0	3.72
0.518	740
1.07	933
1.54	255
2.05	184
3.04	143
4.01	109
5.95	93.1
8.02	73.4

DOSING INFORMATION:

IV Infusion
Dose = 52 mg Infusion time = 1 h

NONCOMPARTMENTAL ANALYSIS OF DATA:

AUC(0-t) =	1.698	μg-h/mL
AUC(0-inf) =	2.337	μg-h/mL
AUMC =	14.66	μg-h^2/mL
MRT =	5.774	h
CL =	22.25	L/h
K(z) =	0.115	1/h
t 1/2 =	6.035	h
Vss =	128.5	L

C(0) bolus = 0. μg/mL

==

ONE-COMPARTMENT SIMULATION LINEAR MODEL:

==

CL =	1.565	L/h	CV= 114.12%
Vd =	54.99	L	CV= 94.57%
CL/kg=	0.026	L/h/kg	CV= 114.12%
Vd/kg=	0.917	L/kg	CV= 94.57%
K10 =	0.028	1/h	CV= 148.21%
Tlag =	0.835	h	CV= 149.41%
Ka =	6.052	1/h	CV= 0.29%
F =	21.95	%	CV= 149.41%
Cmax =	0.018	ug/mL/mg Dose	
t 1/2 =	24.36	h	CV= 148.21%
AUC =	2.337	µg-h/mL	CV= 187.06%
AUMC =	1184.1	µg-h^2/mL	CV= 187.06%
MRT =	35.14	h	CV= 148.21%
R^2 =	-0.6267		

Akaike Information Criterion (AIC) =
(#Pts) * Log(Obj) + 2(#Parameters)
= 5.1092

Schwarz Criterion (SC) =
(#Pts) * Log(Obj) + (#Parameters)*(Log(#Pts))
= 10.3318

Optimization
time: 4.890625 sec
Total simulations: 2784

Weighted sum of squared errors = 7.9224E-1
Weighting: 1/Yhat^2

PREDICTED AND OBSERVED VALUES

Time	Pred	Obs	Residual
(h)	(ng/mL)	(ng/mL)	(ng/mL)
0	0	1	-1
0.875	164.046	164	0.046
1.97	741.432	1218	-476.568
3.67	707.174	760	-52.826
6.4	654.31	544	110.31
8.43	617.58	269	348.58
9.79	594.133	216	378.133
11.9	559.505	243	316.505
24.2	394.255	89.5	304.755
48.1	199.699	31	168.699
72.1	100.864	16.9	83.964
168	6.583	7.58	-0.997
0	0	3.72	-3.72
0.518	486.234	740	-253.766
1.07	930.447	933	-2.553
1.54	918.084	255	663.084
2.05	904.855	184	720.855
3.04	879.716	143	736.716
4.01	855.763	109	746.763
5.95	809.795	93.1	716.695
8.02	763.467	73.4	690.067

==

TWO-COMPARTMENT SIMULATION LINEAR MODEL:

==

CL =	9.414	L/h	CV= 55.54%
Vc =	22.52	L	CV= 122.47%
CL2 =	32.78	L/h	CV= 79.3%
V2 =	305.6	L	CV= 50.19%
CL/kg=	0.157	L/h/kg	CV= 55.54%
Vc/kg=	0.375	L/kg	CV= 122.47%
CL2/kg=	0.546	L/h/kg	CV= 79.3%
V2/kg=	5.093	L/kg	CV= 50.19%

K10 =	0.418	1/h	CV= 134.47%
K12 =	1.456	1/h	CV= 145.9%
K21 =	0.107	1/h	CV= 93.85%

Tlag =	0.8196	h	CV= 68.98%
Ka =	0.475	1/h	CV= 169.3%
F =	78.05	%	CV= 68.98%

Cmax = 0.021 ug/mL/mg Dose
t 1/2 = 30.27 h CV= 0.0%
C* = 2.309 μg/mL

R^2 = 0.9582

Akaike Information Criterion (AIC) =
(#Pts) * Log(Obj) + 2(#Parameters)
= -24.954

Schwarz Criterion (SC) =
(#Pts) * Log(Obj) + (#Parameters)*(Log(#Pts))
= -17.6433

Optimization time: 1.8125 sec.
Total simulations: 1468

Weighted sum of squared errors = 1.5959E-1
Weighting: 1/Yhat^2

PREDICTED AND OBSERVED VALUES

Time	Pred	Obs	Residual
(h)	(ng/mL)	(ng/mL)	(ng/mL)
0	0	1	-1
0.875	163.325	164	-0.675
1.97	1081.382	1218	-136.618
3.67	722.369	760	-37.631
6.4	388.991	544	-155.009
8.43	301.474	269	32.474
9.79	271.532	216	55.532
11.9	244.444	243	1.444
24.2	177.704	89.5	88.204
48.1	102.781	31	71.781
72.1	59.32	16.9	42.42
168	6.597	7.58	-0.983
0	0	3.72	-3.72
0.518	770.716	740	30.716
1.07	944.03	933	11.03
1.54	434.797	255	179.797
2.05	221.115	184	37.115

3.04	112.824	143	-30.176
4.01	95.57	109	-13.43
5.95	88.923	93.1	-4.177
8.02	84.75	73.4	11.35

==

THREE-COMPARTMENT SIMULATION LINEAR MODEL:

==

CL =	14.86	L/h	CV= 24.37%
Vc =	17.2	L	CV= 58.11%
CL2 =	31.13	L/h	CV= 45.1%
V2 =	118.1	L	CV= 47.92%
CL3 =	6.005	L/h	CV= 55.58%
V3=	429.8	L	CV= 0.22%
CL/kg=	0.248	L/h/kg	CV= 24.37%
Vc/kg=	0.287	L/kg	CV= 58.11%
CL2/kg=	0.519	L/h/kg	CV= 45.1%
V2/kg=	1.969	L/kg	CV= 47.92%
CL3/kg=	0.1	L/h/kg	CV= 55.58%
V3/kg=	7.163	L/kg	CV= 0.22%
K10 =	0.864	1/h	CV= 63.02%
K12 =	1.81	1/h	CV= 73.56%
K21 =	0.264	1/h	CV= 65.8%
K13 =	0.349	1/h	CV= 80.41%
K31 =	0.014	1/h	CV= 55.58%
Tlag=	0.843	h	CV= 28.89%
Ka=	0.534	1/h	CV= 72.73%
F=	90.12	%	CV= 28.89%
Cmax =	0.02	ug/mL/mg Dose	
C* =	3.023	ug/mL	
t 1/2 =	71.65	h	CV= 0.0%
R^2 =	0.9851		

Akaike Information Criterion (AIC) =
(#Pts) * Log(Obj) + 2(#Parameters)
= -60.3484

Schwarz Criterion (SC) =
(#Pts) * Log(Obj) + (#Parameters)*(Log(#Pts))
= -50.9467

Optimization
time: 4.585938 sec.
Total simulations: 3176

Weighted sum of squared errors = 2.3168E-2
Weighting: 1/Yhat^2

PREDICTED AND OBSERVED VALUES

**Time**	**Pred**	**Obs**	**Residual**
(h)	(ng/mL)	(ng/mL)	(ng/mL)
0	0	1	-1
0.875	164.012	164	0.012
1.97	1219.883	1218	1.883
3.67	773.396	760	13.396
6.4	447.061	544	-96.939
8.43	338.082	269	69.082
9.79	289.508	216	73.508
11.9	233.823	243	-9.177
24.2	85.193	89.5	-4.307
48.1	28.419	31	-2.581
72.1	19.203	16.9	2.303
168	7.438	7.58	-0.142
0	0	3.72	-3.72
0.518	806.999	740	66.999
1.07	841.156	933	-91.844
1.54	299.678	255	44.678
2.05	169.059	184	-14.941
3.04	127.537	143	-15.463
4.01	115.212	109	6.212
5.95	96.011	93.1	2.911
8.02	79.363	73.4	5.963

****

AIC indicates three-compartment model is preferred.
SC indicates three-compartment model is preferred.

****

Search Method: Hooke & Jeeves Pattern Search
Weighting: 1/Yhat^2
