# Supplementary material for: Indirect Modeling of Post-Prandial Intestinal Lymphatic Uptake of Halofantrine Using PBPK Approaches: Limitations and Implications
Source: Pharmaceutics. 2025 Sep 22;17(9):1228. doi: 10.3390/pharmaceutics17091228 (PMC12473779; doi:10.3390/pharmaceutics17091228)
Supplement: Supplementary file 1 [file pharmaceutics-17-01228-s001.zip › Supplementary Files S3.pdf]

## Supplementary Materials

Molecular Dynamics (MD) Simulations of Halofantrine

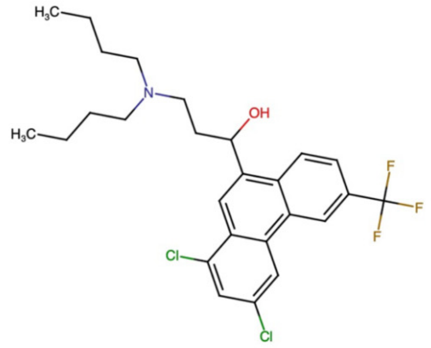


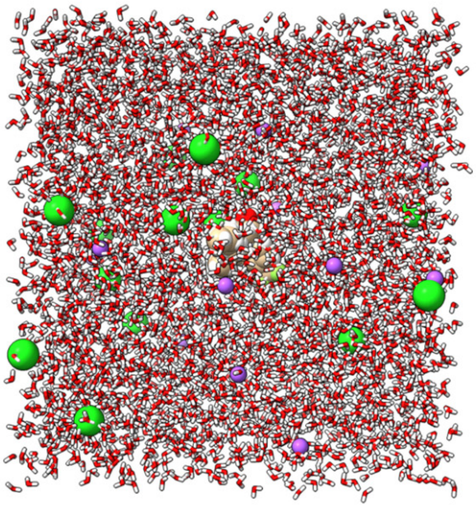

**(a)** **(b)**

**Figure S1.** (a) 2D structure of halofantrine (b) and water molecules with ions (sodium & chloride) surrounding halofantrine as used in the MD simulations to replicate the physiological solvation environment.

**Results of Halofantrine MD simulations:**

Molecular dynamics (MD) simulations of halofantrine were conducted over 1000 ns to assess its stability and conformational behavior using root-mean-square deviation (RMSD) analysis. The protonated form showed moderate stability, with RMSD values ranging from 3 to 4 Å and peaking at approximately 4.5 Å, indicating limited conformational fluctuations. In contrast, the unprotonated form was more stable overall, with RMSD values fluctuating between 1 and 3 Å. Notably, this state exhibited greater conformational diversity, sampling distinct structures around 100 ns, 300 ns, 500 ns, 650 ns, and between 780–950 ns. Several of these conformations reverted to an RMSD of ~1 Å relative to the initial structure, suggesting repeated access to low-RMSD states (Figure S2). Clustering analysis revealed approximately 10 dominant conformations for the unprotonated form and 8 for the protonated form, as summarized in Table S1. Superimposition of the top representative conformations from both states (Figure S3) illustrated the range of structural changes captured in the RMSD analysis, highlighting the ligand's conformational flexibility throughout the simulation.

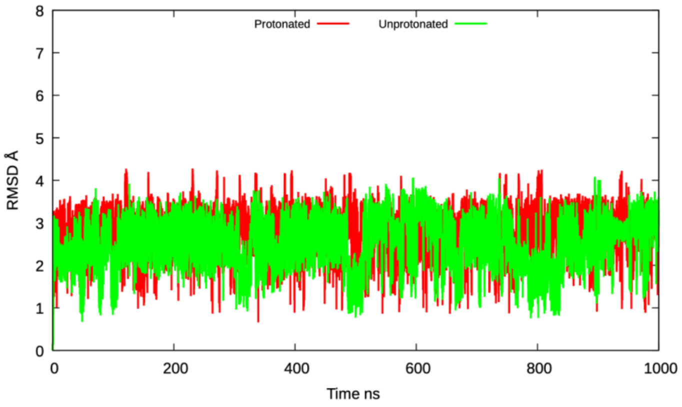


**Figure S2.** RMSD of protonated and unprotonated forms of halofantrine during 1000ns MD simulations.

**Table S1.** Percent of all conformations in dominant cluster in unprotonated & protonated systems of halofantrine.

| Conformations in cluster | Population percent in the unprotonated species | Population percent in the protonated species |
|---|---|---|
| Representative #1 | 25% | 32% |
| Representative #2 | 20% | 32% |
| Representative #3 | 12% | 10% |
| Representative #4 | 10% | 8% |
| Representative #5 | 9% | 8% |
| Representative #6 | 7% | 7% |
| Representative #7 | 7% | 2% |
| Representative #8 | 5% | 1% |
| Representative #9 | 3% | NA |
| Representative #10 | 2% | NA |

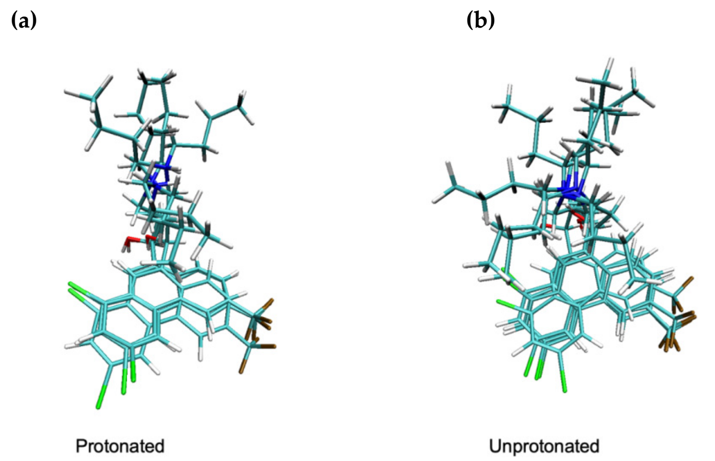


**Figure S3.** Superimposition of the most dominant confirmations of (a) protonated and (b) unprotonated states of halofantrine.

Figure S4 illustrates the number of water molecules within a 5 Å cutoff from the upper and lower sides of halofantrine over the course of the simulation. In the protonated state, the presence of a protonated nitrogen and a hydroxyl group, both capable of forming polar or charged interactions led to enhanced water association. Approximately 8 to 15 water molecules were consistently observed near the upper side, while around 2 were present on the lower side. In contrast, the unprotonated state retained only the hydroxyl group for polar interactions. This resulted in 5 to 14 water molecules associating with the upper side, with a noticeable decrease to around 4 molecules at 950 ns. On the lower side, hydration remained limited, with water molecule counts fluctuating between 0 and 2, indicating reduced interaction compared to the upper region.

**(a)** **(b)**

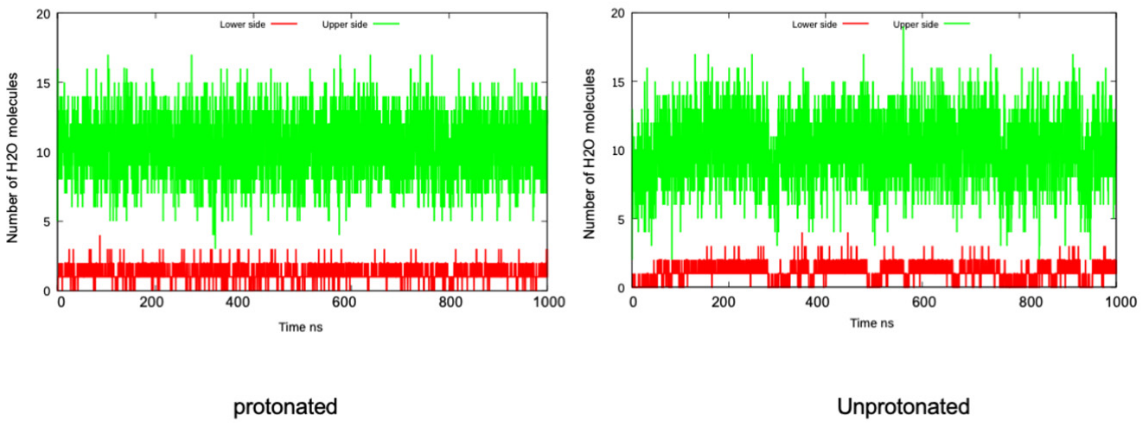


**Figure S4**. Number of water molecules within 5 Å on the upper and lower sides of halofantrine during the entire simulation for (a) the protonated and (b) the unprotonated species.
